# A case study on breastfeeding education in Lebanon’s public medical school: exploring the potential role of social networks in medical education

**DOI:** 10.1080/10872981.2018.1527629

**Published:** 2018-10-09

**Authors:** Sara Moukarzel, Christoforos Mamas, Melissa F. Warstadt, Lars Bode, Antoine Farhat, Antoine Abi Abboud, Alan J Daly

**Affiliations:** a Larsson-Rosenquist Foundation Mother-Milk-Infant Center of Research Excellence, University of California San Diego, La Jolla, CA, USA; b Department of Education Studies, University of California San Diego, La Jolla, CA, USA; c Faculty of Nursing and Health Sciences, Notre Dame University, Zouk Mosbeh, Lebanon; d School of Medicine, Lebanese University, Hadath, Lebanon

**Keywords:** Breastfeeding, medical education, attitudes, beliefs, knowledge assessment, social network, Lebanon

## Abstract

**Background**: Limited knowledge, negative beliefs, and lack of sufficient breastfeeding promotion and support by physicians contribute to global suboptimal breastfeeding rates. Formal medical education is well-known to influence future physicians’ knowledge, beliefs, and medical practice. However, less understood is the influence of social networks and processes on the exchange and diffusion of knowledge and practices related to breastfeeding. **Objectives**: We selected the underserved and under-supported public medical school in Lebanon to examine the social side of medical education. Our objectives were to assess knowledge, beliefs, and self-efficacy related to breastfeeding promotion and support among interns and residents. We also examined the social ecosystem surrounding these students concerning the exchange of breastfeeding knowledge. **Design**: All data were collected during one study visit per participant. First, an interview-administered structured survey was used to assess beliefs, perceived knowledge, basic breastfeeding knowledge, and self-efficacy related to breastfeeding among *n *= 70 medical interns and residents. Then, social network data were collected during a semi-structured interview and analyzed using an ego-network approach. All interviews were voice-recorded, transcribed, coded, and thematically analyzed. Descriptive statistics were used to analyze quantitative survey and social network results. **Results**: Although interns and residents had positive beliefs about breastfeeding benefits, they had limited knowledge and low self-efficacy related to the psychosocial and clinical aspects of breastfeeding promotion and support. They did not seem to have a well-connected professional network around breastfeeding knowledge and practices. Several tended to rely on their informal/non-professional network, such as their mothers, partners, and sisters, for knowledge and practice. **Conclusions**: Our work using breastfeeding as an exemplary case suggests there is a role for better attending to the beliefs of medical students as well as to the social side of medical education. Future studies can use social network theory to help identify and address influences on medical education outcomes.

## Introduction

The role of medical education extends beyond knowledge dissemination only to also include attitudinal and behavioral change related to medical practice [1–3]. The large influence of formal classroom education and clinical training on future physicians’ knowledge, beliefs, and behavior is well-known. However, as social beings, humans are also largely influenced by the relationships they build within their wider social systems (families, friendship groups, schools, workplaces) [4–6]. Relevant to the medical education context, social relationships may influence academic performance, attitudes towards medical practice issues, and learning during clinical rotations [4]. Yet, our understanding of the social processes underlying trainees’ medical education outcomes remains limited. In this paper, we use *knowledge around breastfeeding among interns and residents* as an exemplar case to better elucidate the role of social networks in medical education.

The health and survival benefits of breastfeeding for both mother and child are well-documented in the scientific literature and have been widely disseminated in guidelines by the World Health Organization and others [7–9]. However, suboptimal uptake of this knowledge by physicians is not uncommon [10,11], and insufficient breastfeeding promotion and support is one contributor to low breastfeeding exclusivity, initiation, and continuation rates [9,12–18]. Physicians may be particularly influential in communities where availability of and access to lactation consultants is limiting or when physicians are culturally considered as the most trusted experts compared with other health professionals [19,20].

Barriers against breastfeeding promotion and support by physicians broadly and by pediatricians, gynecologists, and family physicians specifically are complex and setting-specific. Around the globe, limited knowledge, negative beliefs, and lack of interest in the topic of breastfeeding have been reported [15,18,19,21–23]. It may be possible to overcome these barriers in part through early interventions beginning in undergraduate medical schools and extending to relevant residency programs [10,11]. One provocative idea is to use social network theory in the medical education environment to understand and influence the beliefs and behavior of future physicians before they become well-established in their own clinical workflow [1,4,24].

This aforementioned approach is what may be described as the ‘social side’ of change, knowledge uptake, and implementation of new practices that are the direct result of physicians’ social networks and connections within them. For our initial foray into this work, we selected a developing nation (Lebanon) and its only *public* medical school for three core reasons. First off, public schools in these settings are typically underserved and under-supported: Largely due to limited government funding support, factors such as overcrowded classes and limited number of instructors may contribute to students not receiving similar learning opportunities to those from well-funded private institutions. Second, medical schools in developing nations are serving an enormous fraction of the population, and yet they are under researched and do not receive as much attention in the larger medical education literature. Third, physicians in Lebanon are known to be highly influential on patient health practices, and suboptimal breastfeeding rates particularly in developing nations may have drastic consequences on maternal and infant health [8,17,18,21]. Indeed, approximately 38% of infants only are exclusively breastfed during the first month of life and around 2% at 6 months of age in Lebanon [25,26]. To put this into perspective, this is strikingly lower than ~ 30%, the estimated average exclusive breastfeeding rate at 6 months for infants in countries around the globe which are in the same country income group as Lebanon (upper-middle income) [8]. Additionally, the training hospitals affiliated with the Lebanese public medical school are not designated as Baby-Friendly and do not have policies that enforce the implementation of the majority of the 10 Steps to Successful Breastfeeding [27]. All of these factors combine to make Lebanon a unique and compelling case to examine the social side of medical education.

Social network theory provides a conceptual framework for understanding how people or organizations interact with others within their environment and how these social interactions affect a wide range of outcomes (e.g., academic performance, motivation, knowledge uptake, beliefs). It is well-known that social relationships and the resultant social interactions, which are collectively described as a social network, have value [28–30]. This is because social networks have the potential to impede or to provide access to resources (e.g., knowledge and skills) that can be exchanged, borrowed, and leveraged to facilitate achieving goals [28–30]. Indeed, there is a growing empirical base around social network theory in a variety of disciplines including education [4,5].

In our own work over the last 1.5 years, we have been using this systematic approach to understand and address potential supports and constraints of breastfeeding promotion and support in a group of medical interns and residents in a large-scale research project. In this paper, we report findings from the first pilot phase of our formative research which includes assessment of knowledge, beliefs, and self-efficacy related to breastfeeding promotion and support. We also describe the social network of the Lebanese cohort around breastfeeding knowledge exchange.

## Methods

### Sample

This study includes a sample of medical interns and residents who were enrolled in 2016–2017 at the only *public* medical university in Lebanon. The university adopts the French model of medical education: undergraduate medical education consists of a 7-year program after a baccalaureate degree compared with a 4-year program after an undergraduate degree in the American model. Interns refer to medical students rotating in clinical clerkships during their sixth and seventh year, which is equivalent to Med III and Med IV in American universities. Similar to the American model, duration of residency programs [number of postgraduate years (PGY)] varies by specialty and ranges between 3 and 7 years. In Lebanon, there are seven registered medical schools, two of which implement the French model, three implement the American model, and one incorporates the British model (6-year program) [31].

Participants were recruited at two affiliated hospitals (hospital A and hospital B) in Mount Lebanon (Supplemental Figure 1) at which the interns and residents were training. 70 participants completed the study (*n *= 55 at hospital A and *n *= 15 at hospital B; response rate of 90%). Responses did not significantly differ by hospital setting, and results are, therefore, reported for the cohort as a whole. All participants were Lebanese and unmarried without children except for one PGY5-level female resident specializing in obstetrics and gynecology (OB/GYN); other participant’s characteristics are summarized in Table 1 and Supplementary Figure 2. Written informed consent was obtained, and all aspects of the study were approved by the Institutional Review Boards at the University of California San Diego and affiliated Lebanese School of Medicine.

**Table 1.** Participant characteristics.

	Interns		Residents
	Med III *n *= 34	Med IV *n *= 14	*n *= 22
Age, years	23.3 ± 0.99	24.0 ± 0.73	26.8 ± 1.22
Gender, female	20 (59)	8 (57)	11 (50)
Area of residence* Akkar North Lebanon Mount Lebanon Beirut South Lebanon Baalbek-Hermel Beqaa Nabatieh	0 8 (23.5) 18 (53) 0 5 (14.7) 2 (5.8) 1 [3] 0	2 (14.3) 2 (14.3) 5 (25.7) 2 (14.3) 1 (7.1) 1 (7.1) 0 1 (7.1)	0 4 (18.2) 15 (68.2) 0 0 0 3 (13.6) 0

Values are mean ± SD or *n* (%) for continuous or categorical variables respectively. *A map of Lebanon illustrating its 8 governorates (equivalent to states or provinces) can be found in Supplementary Figure 1.

### Data collection

All data for each participant were collected during one study visit conducted in a private office at the hospital where the participant was recruited. Each visit lasted 60–90 min. First, an interview-administered structured questionnaire was used to collect demographic information and to assess beliefs related to benefits of breastfeeding, perceived knowledge about breastfeeding, and self-efficacy related to providing breastfeeding support. The questionnaire was completed by the participant manually with guidance from the interviewer. To assess beliefs related to breastfeeding benefits, participants rated their approval level (six options from ‘strongly disagree’ to ‘strongly agree’) to the following three statements: I believe that human milk provides the term infant with adequate nutrition; I believe that exclusively breastfed infants have fewer gastrointestinal infections, respiratory illnesses, eczema, and/or allergic reactions than formula-fed infants; I believe that the longer women breastfeed, the more they are protected against breast cancer. Similarly, participants rated their level of agreement on statements that assessed either perceived knowledge or self-efficacy related to breastfeeding. To assess the reliability of the respective two subscales, an Exploratory Factor Analysis (EFA) with principal component extraction was conducted on each. The two subscales were analyzed separately, with consideration of the item themes. The first subscale, Perceived Knowledge, included seven items; the second subscale, Self-Efficacy, included nine items. For both tests, Varimax (orthogonal) rotation of the items was utilized, given the uniqueness of the items and low correlations. The factorability and reliability of both subscales were assessed. The Kaiser–Meyer–Olkin test of the sample indicated that the items could be factorable (KMO = 0.723 and 0.721, respectively) and the Bartlett’s Test of Sphericity was significant for both (*P *< 0.001). Cronbach’s Alpha demonstrated fairly strong reliability across items (alpha = 0.721 and 0.829, respectively).

The Perceived Knowledge scale is factored into two components: 1) Perceived Knowledge of Anatomy and Physiology and 2) Perceived Knowledge of Benefits and Recommendations. Examples of items included, *I know the anatomy of the lactating breast* and *I know common physiological problems some women face to initiate or continue breastfeeding*. The Self-Efficacy scale is factored into two components: 1) Efficacy of Providing Psycho-Social Support and 2) Efficacy of Counseling about Breastfeeding. Examples of items included, *I know common psycho-social problems some women face related to breastfeeding* and *I am confident that I can explain the benefits and potential challenges of breastfeeding in a way that the patient understands*. These results indicate that the items making up the subscales are reliably being answered in a predictable pattern. The purpose of the EFA was not to validate a scale to use on another sample, but to assess the reliability of these items for the current study only.

**Figure 1.**
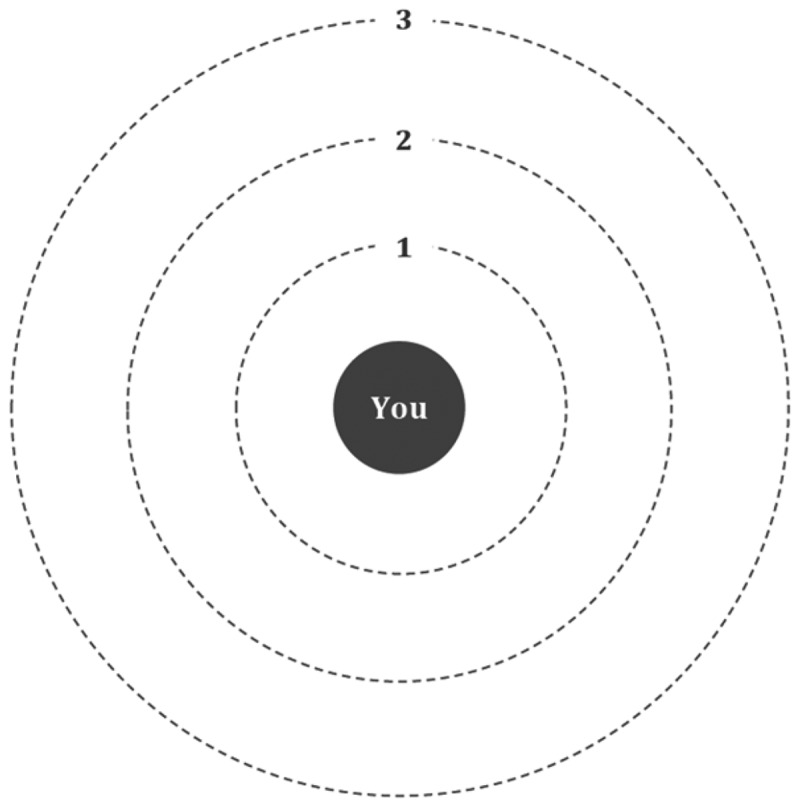
Concentric circles used to collect ego-net data.

After completing the structured survey, social network data were collected using an ego-network approach [32,33]. The participant, referred to as ego, was presented with concentric circles (Figure 1) and was asked to write the names of alters (people with whom the ego talked to over the last 6 months to learn about any aspect of breastfeeding support) on the three circles based on ascending order of frequency of communication. Alters with whom the ego mostly communicated with were placed on circle 1 and those with whom the ego less or least communicated with (but still communicated) were placed on circles 2 and 3, respectively. Since no comparative data are available related to the social network of participants around advice about their medical practice in general, we also asked each ego the question ‘to whom did you talk to over the last 6 months to get advice related to your medical practice’. We provided participants with clarifying examples such as ‘advice related to which diagnostic tests to run or which medications to prescribe’. As participants were completing this task, they were probed to discuss why they chose their particular alters.

Finally, after collecting the social network data, the Wellstart International 28-point multiple choice test was administered to assess basic breastfeeding knowledge, defined as Level I knowledge [34]. Out of the three levels of knowledge, Level I is the most basic and is tailored to all healthcare professionals regardless of whether they specifically provide care for breastfeeding mothers and infants. Level I knowledge encompasses basic understanding of the scientific basis behind promoting and supporting breastfeeding, the physiology and basics of clinical management of lactation for healthy mother-infant dyads, and the societal influences on breastfeeding and breastfeeding promotion.

### Data analysis

Descriptive statistics (SPSS version 25 for Mac) were used to analyze survey responses as well as the quantitative social network data (i.e., number of alters per participant and characteristics of alters such as professional affiliation or relationship to ego) [32,33]. Qualitative social network data (i.e., reasons for choosing particular alters) were transcribed, coded, and thematically analyzed using MAXQDA (VERBI GmbH Berlin, 2018). The knowledge assessment tests were corrected by two researchers and cross-checked for accuracy; results are reported using descriptive statistics. Given the limited sample size per level of training particularly among residents, comparisons across levels of residency training are only considered exploratory.

## Results

### Breastfeeding knowledge assessment

Test scores, as percentage of correct answers, were skewed with higher mean than median and ranged between 25.0 and 78.6% (Table 2). Only four participants received a satisfactory test score, previously and arbitrarily set at > 70% [22,35]; These were two Med III-level interns each scoring 71.4%: one PGY2-level resident in pediatrics scoring 78.6% and one PGY5-level resident in OB/GYN scoring 78.6%. When stratified by level of training, test scores were normally distributed and mean test scores were significantly higher among residents than interns (*P *= 0.009). No significant difference in mean scores was found between Med III- and Med IV-level interns (*P *= 0.068). Mean test scores among residents did not significantly differ by level of training (*P *= 0.131), although this may be explained in part by the difference in sample size across groups. The subset of participants who were specializing/considering to specialize in OB/GYN or pediatrics (*n *= 20) did not score significantly higher than others (58.7 ± 11; 55.4 ± 10.1, *P *= 0.233).

**Table 2.** Test scores as % correct answers on the breastfeeding knowledge assessment test.

	Sample size	Mean	SD	Median	Min	Max
**All**	69	54.3	10.7	53.6	25.0	78.6
**Interns** Med III Med IV	47 34 13	52.0 50.2 56.6	10.7 11.3 7.54	53.6 50 53.6	25.0 25.0 42.9	71.4 71.4 67.9
**Residents** PGY1 PGY2 PGY3 PGY5	22 11 7 2 2	59.1 57.5 57.1 60.7 73.2	9.14 6.86 11.1 5.08 7.60	57.1 57.5 57.1 60.7 73.2	42.7 50 42.9 57.1 67.9	78.6 67.9 78.6 64.3 78.6

Consistent with the quantitative knowledge assessment results, which indicate suboptimal knowledge related to breastfeeding, one Med IV-level student who captures the general opinion noted, ‘we should know more because our knowledge about breastfeeding is still not enough. All what we know is that breast milk is way better (than infant formula). But people ask us, interns or residents, about these topics: how should breastfeeding be like? When should I stop breastfeeding… We should know more to explain more things to the patient.’ A second female student shared, ‘I honestly haven’t read a lot of comparative studies with formula-fed babies and breastfed babies. But of course, if I read and learn that there is a huge difference between the two, then I would have no problem (to promote and support breastfeeding).’ Coupling the quantitative results with the qualitative analysis portrays an overall lack of knowledge in this space and the need for additional information.

### Beliefs, perceived knowledge, and self-efficacy

All participants agreed to various extents that human milk is an adequate nutrition source and is immune-protective to the infant. The majority (88.5%) also agreed that breastfeeding protects against maternal breast cancer. Belief levels did not differ significantly between interns and residents. Mean scores on perceived knowledge and self-efficacy factors ranged between 64 and 74% of six, the maximum possible score, but ranged widely between 2 and 6 across participants (Supplementary Table 1). Mean scores were not significantly different between interns and residents, except for higher self-efficacy among residents related to counseling about breastfeeding. The subset of participants who are specializing/considering to specialize in OB/GYN or pediatrics scored significantly higher on factors [1,2], and [4] compared with others (Table 3). Interestingly, scores on perceived knowledge and self-efficacy for factor [1,2] and [3] were significantly associated with the knowledge assessment test scores only among participants who are specializing/considering to specialize in OB/GYN or pediatrics (Table 4). Test scores did not seem to increase with increasing level of perceived knowledge and self-efficacy among the rest.

**Table 3.** Mean scores on perceived knowledge and self-efficacy scales by whether (YES) or not (NO) participants are specializing/considering to specialize in OB/GYN or pediatrics.

	YES (*n *= 20)	NO (*n *= 49)	*P*-value
**Perceived knowledge** Factor 1: Anatomy and physiology Factor 2: Benefits of breastfeeding	4.29 ± 0.90 4.53 ± 0.84	3.89 ± 0.67 3.90 ± 0.94	0.047 0.012
**Self-efficacy** Factor 3: Providing psycho-social support Factor 4: Counseling about breastfeeding	4.23 ± 0.87 4.25 ± 0.74	4.12 ± 0.96 3.83 ± 0.90	0.662 0.024

Values are mean ± SD. Maximum mean score per factor is 6. Differences between groups are analyzed by independent sample *t*-test.

**Table 4.** Correlations between actual knowledge and factors measuring perceived knowledge and self-efficacy by whether (YES) or not (NO) participants are specializing/considering to specialize in OB/GYN or pediatrics.

	Factor 1	Factor 2	Factor 3	Factor 4
YES, *n *= 20	r = 0.452	r = 0.474	r = 0.660	r = 0.441
	*P *= 0.046	*P *= 0.034	*P *= 0.002	*P *= 0.052
NO, *n *= 49	r = 0.162	r = −0.162	r = -0.613	r = −0.143
	*P *= 0.270	*P *= 0.273	*P *= 0.269	*P *= 0.332

Correlations analyzed by Pearson’s Correlation. Factor 1: Perceived knowledge of anatomy and physiology of breastfeeding. Factor 2: Perceived knowledge of benefits of breastfeeding. Factor 3: Efficacy of providing psycho-social support and Factor 4: Efficacy of counseling about breastfeeding

### Social networks around breastfeeding knowledge

**Figure 2.**
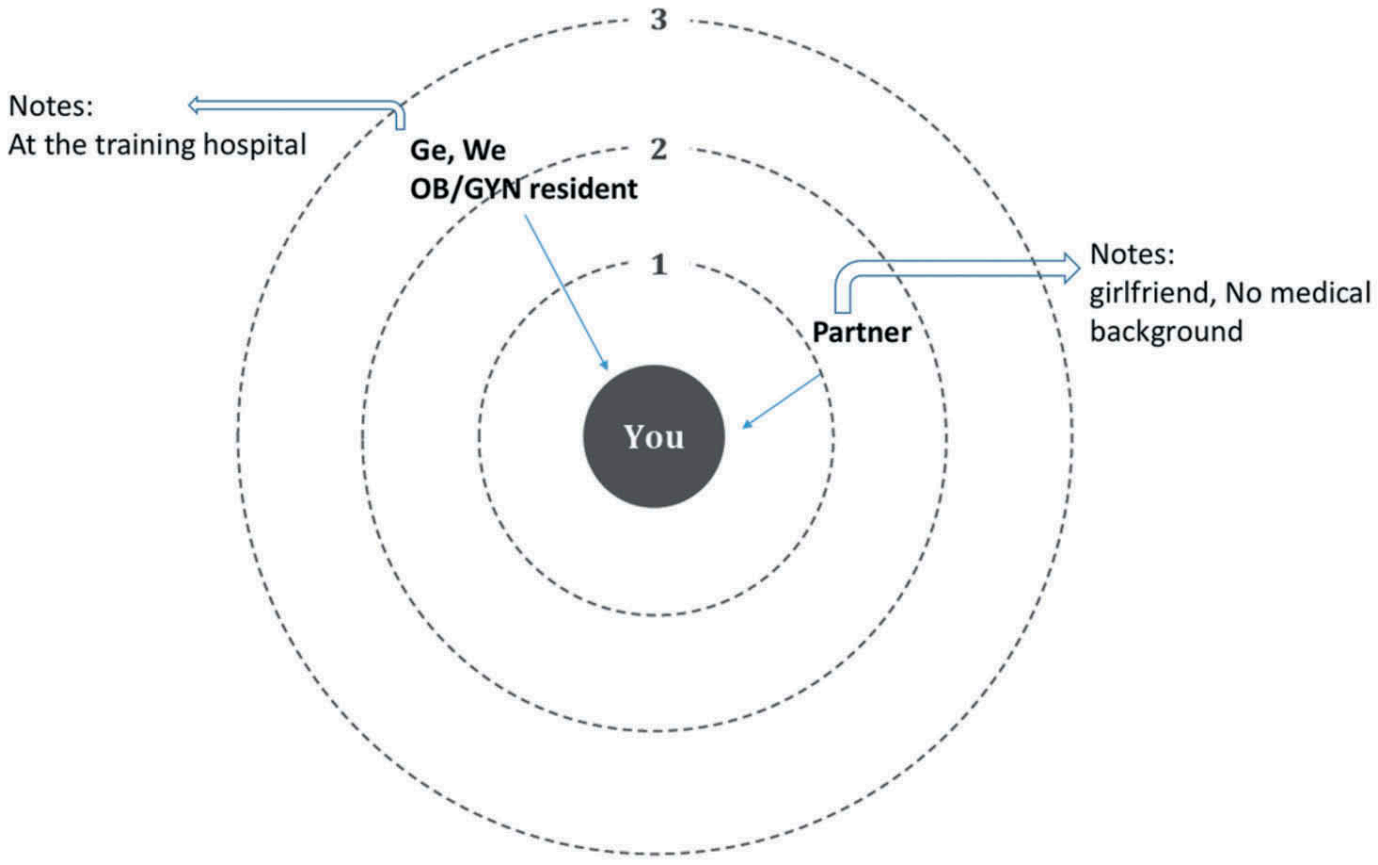
Example showing one participant reported learning about breastfeeding support over the last 5 months from his partner (girlfriend) and from one OB/GYN resident at the hospital.

Only 24% of the participants reported ever talking to someone to learn about any aspect of breastfeeding support in the last 6 months. We, therefore, asked the remaining participants to describe with whom they *would* talk if they want to learn today. Because none of the findings differed between those who did talk or did not talk about breastfeeding support, as well as between interns and residents, we just report the pooled results. Figure 2 shows one example of how the raw ego-net data looked like for one participant.

On average, participants talked/would talk to only two individuals (alters) [2 ± 1.3; median (range):2 (0–9)] to learn about breastfeeding support compared with six alters to learn about other topics [6 ± 4.3; median (range): 5 (1–24); *P *< 0.001). Alters at the university and affiliated hospitals for breastfeeding advice included (in decreasing order of frequency) OB/GYN attending physicians, attending pediatricians, neonatology fellows, OB/GYN residents, residents in pediatrics, and Med IV-level as well as Med III-level interns. Intriguingly, *n *= 15 participants reported talking/would talk to learn about breastfeeding with individuals who do not have any medical education background. These included (in decreasing order of frequency) the participants’ mothers, sisters, and a female partner, who were not mentioned when asked about advice related to medical practice. Additionally, we identified 11 female participants who learned/would learn about breastfeeding support from healthcare professionals not affiliated with the university and training hospitals. These included participants’ personal OB/GYN, mother’s OB/GYN, personal pediatrician, and midwife with whom they have family or friendship ties.

The total number of alters did not significantly vary with demographic characteristics (including gender), specialty, belief levels, perceived and actual knowledge scores, or self-efficacy scores. We,, therefore explored whether personal traits and informal social connections explained participants’ choice of alters by thematically analyzing the content of interview transcript sections pertaining to the answers to our open-ended question ‘Why did you chose this/these alter(s) to learn about breastfeeding’. While some alters were chosen because they were simply at a higher level in the hierarchal communication structure at the hospital compared with the ego, we found four common reasons related to personal traits and informal social connections. Due to space limitations, a few representative quotes for each theme are listed in Table 5. First, some alters were chosen because they were perceived as effective teachers who love to teach. Another common reason was that alters and egos have close friendship. Additionally, some alters were chosen because they were perceived as knowledgeable or experienced in their medical specialties. Finally, some alters were chosen because they were the ego’s family members with personal breastfeeding experience. Therefore, the social network of our study participants related to knowledge exchange around breastfeeding seems to be influenced in part by personal relationships and seems to extend beyond formal education and training within the medical school and the hospital settings. These preliminary results may have important implications to the quality of knowledge (evidence-based information, misconceptions, and myths) and self-efficacy around breastfeeding promotion and support.

**Table 5.** Common reasons for choosing particular alters.

Common theme	Direct Quotations as examples
Alters perceived as effective teachers who love to teach	‘He [alter, fellow] loves to teach us. He could be able to finish his duties in the hospital ward within 2 min (metaphor meaning ‘quickly’). Instead, he stays with us for 1.5 h just to talk about what we should do or what we should have been done.’ ‘They [alters, residents] answer our questions quickly, they have discussions with us, they teach us, they tell us “why something is correct” or “why something is wrong”. And they are very compassionate’. ‘They [alters, physicians] are very down to earth. There are some physicians that just talk to you to get medical updates about their patients and that’s it. Bye! With others, we discuss the (medical) case and talk about the different potential diagnoses and why we thought about that, why we chose this treatment’. ‘The OB/GYN resident is very nice. He loves to teach. One time, we were having lunch at the cafeteria, and he casually started giving us [interns] a talk [about breastfeeding]. I was like wow’.
Alters have close friendship with ego	‘We [alters and ego; residents] became close friends. I feel comfortable around them, and they feel comfortable around me. And they are very good (ethical) guys’. ‘I wouldn’t go to an attending physician (to learn about breastfeeding support). I would go to my friends (interns at hospital). I won’t talk to the attending physician out of nowhere. I am more used to my friends. And the information would still be fresh in their memories’. ‘He [alter, intern] doesn’t complicate things. I feel like his personality is like mine. He is like my brother. I believe there’s a solution to everything (every problem) and he believes that too’.
Alters perceived as knowledgeable or experienced in their medical fields	‘She [alter, resident] has so much information. She knows the (medical) guidelines. Sometimes she corrects the physicians at conferences. Her knowledge is so up-to-date’. ‘He [alter, resident] directly responds to me, and he quickly knows the answers (to my questions) and if he doesn’t know, he directly looks the information up’. ‘He [alter, OB/GYN physician] has read everything related to obstetrics and gynecology: Infertility, oncology, basics, physiology. He will never tell you any information that you would find incorrect. Never. Also, he has lots of experience, around 30 years, and he is always up to date. He reads for 2 h every day’.
Alters as family members with personal breastfeeding experiences	(I learn about best feeding practices) from my sisters. When they breastfed and gave formula at the same time, their infants gained more weight than breastfeeding alone. The IgA and antibodies that are needed are taken from breastfeeding. I encourage her (female patient) since day 1 to give mixed feedings (breastfeeding and formula). ‘(I ask) my mother because she breastfed 4 babies so she might know (about breastfeeding challenges and how to address them)’. ‘I was honestly against breastfeeding at first. I used to think that it might be physiologically better if the woman doesn’t breastfeed. Then, they (my sisters) started breastfeeding. I found a lot of advantages. After that, I started reading about all the advantages of breastfeeding. I became like them (pro-breastfeeding)’.

## Discussion

In this study, we identified limited knowledge in breastfeeding basics (i.e., anatomy of the breast, physiology of lactation, clinical management of lactation, WHO recommendations) as a potential individual-level barrier for breastfeeding promotion and support among medical interns and residents at Lebanon’s public medical school. Positive beliefs about the benefits of breastfeeding were common, unlike levels of perceived knowledge and self-efficacy that varied widely across individual trainees. To our knowledge, this is the first study to document limited knowledge and low self-efficacy among future physicians in Lebanon using assessment surveys. Previous interview studies, however, with Lebanese physicians and stakeholders in breastfeeding policy reported barriers consistent with our findings [15,17,18]. While our focus is not on current trainee practices, it is possible that individual-level barriers to promote and support breastfeeding during medical training persist during clinical practice if not addressed [24].

One may argue that improving breastfeeding knowledge and self-efficacy may be more efficient and impactful on improving breastfeeding rates when done through graduate medical education and continued medical education for pediatricians, obstetricians, and gynecologists, and family physicians specifically. However, within the Lebanese context and likely within other Middle Eastern countries as well, physicians are culturally perceived as ‘medical experts in everything’ (quote from one OB/GYN male resident) independent of their areas of specialty. They are often approached informally for medical advice through wide social connections (i.e., friends, family, neighbors, friends of friends). Consistent with previous reports on the influential role physicians have on women’s breastfeeding-related decisions in Lebanon [18,19,21], many of our participants highlighted that ‘buy-in’ from women to initiate and continue breastfeeding may be largely influenced by physicians they trust (*manuscript in preparation*). Therefore, the period during undergraduate and graduate medical education for all areas of specialty may provide a critical window of opportunity to target a broad audience of future physicians for maximized impact on breastfeeding rates in Lebanon.

In addition to individual-level barriers, we identified the lack of a connected social network around evidence-based breastfeeding knowledge as a potential organizational-level barrier. Social network analysis helps gain better understanding of the flow of information around breastfeeding among trainees and their environment. It allows us to visualize social relationships and determine who interacts with whom. This is important because these networks of communication either enhance or impede social capital [29,36,37]. We found that trainees do not typically discuss information around breastfeeding, and if they were to do that, they would not only reach out to medical professionals with assumed evidence-based knowledge (e.g., attending pediatrician or attending gynecologist). They would also reach out to other interns and residents because of their close friendship ties or with friends and family members who have no medical or healthcare-related education background. While close friendship ties and resultant trust among trainees are important resources for social capital, misinformation may be exacerbated without the flow of evidence-based information within the network. Indeed, it has been documented that family members in Lebanon, particularly the breastfeeding mother’s mother, may discourage women to breastfeed [38,39] and may spread misconceptions around breastfeeding. For example, these include: women are biologically incapable of breastfeeding because their mothers were not able to successfully breastfeed them; women are not providing sufficient amounts of milk because their infants are crying; abdominal cramps can be transferred from mother to infant through human milk [38,39]. Of note, the significantly lower number of alters that are sought for advice related to providing breastfeeding support compared with medical advice in general is expected as medical advice in general will encompass a broader range of medical conditions. However, one interesting finding in this case, which needs to be confirmed with a larger sample, is that the majority of participants did not seem to be learning about any aspect of breastfeeding support (at least during the last six months of training and through personal interactions).

Several limitations need to be considered while interpreting our results and need to be addressed in future studies. There were only five pediatrics and OB/GYN residents training at the hospitals during our data collection period, as the two hospitals together only offer 5–7 rotating positions every 6 months. Because of the limited number of residents, our comparative analyses between interns and residents should only be considered exploratory and non-generalizable. Of note, the pediatrics and OB/GYN residency programs at the Lebanese University only offer a handful of positions each year and residents train at hospitals across the country. Future efforts to recruit these residents need to address this potential obstacle. Other studies also need to consider interns and residents at private medical schools which could have different education curricula and training programs compared with the Lebanese University, the only public medical school in Lebanon. This study was not designed to develop or validate scales that assess perceived knowledge and self-efficacy related to breastfeeding. The results of the EFA on the subscales described in the methods section should be considered exploratory in nature and not definitive. These scales were not designed for reproduction on other samples, but simply to understand what was being measured in our case. Future studies should continue to validate these items with larger samples.

This study focused on a cohort within a specific geographical location and focused on breastfeeding promotion and support. However, our research approach has wider implications as to how medical educators may consider overcoming barriers to behavior change in medical education independent of location. Indeed, individual, organizational, and society-level barriers in translating scientific evidence into everyday clinical practice are well-documented for a wide range of health topics other than breastfeeding across the world (e.g., prevention of lifestyle-related chronic diseases) [8–10,40]. Our work suggests that there is a more social side to medical education and some of that education may be happening through informal ties. Applying social network theory can help medical schools explain previously unknown influences on future physicians including the spread of knowledge, beliefs, and behaviors.

Moving forward, our social network analysis allowed us to identify the few influencers who were most commonly reported as having the evidence-based knowledge around breastfeeding, the experience in clinical breastfeeding management, and the passion and dedication to teaching. After confirming our findings in a larger study, our next step to improve knowledge and self-efficacy will be to develop a social network theory-based intervention with the influencers themselves by capitalizing on their roles as agents of change [5]. To our knowledge, it will be the first time a social network-based intervention is used in medical education [4].

In conclusion, this study showed that although a group of interns and residents had positive beliefs about breastfeeding benefits to maternal and infant health, they had limited knowledge and low self-efficacy related to the psychosocial and clinical aspects of breastfeeding promotion and support. They did not seem to have a well-connected professional network around breastfeeding knowledge and practices and several tended to rely on their informal/non-professional network, such as their mothers, partners, and sisters, for knowledge and practice. These results need to be confirmed in larger studies and may have important implications to the quality of knowledge and self-efficacy around breastfeeding promotion and support for both medical education and practice.
