# Supplementary material for: A case study on breastfeeding education in Lebanon’s public medical school: exploring the potential role of social networks in medical education
Source: Med Educ Online. 2018 Oct 9;23(1):1527629. doi: 10.1080/10872981.2018.1527629 (PMC6179049; doi:10.1080/10872981.2018.1527629)
Supplement: Supplemental Material [file ZMEO_A_1527629_SM0837.docx]

**Supplementary Figure 1**. The eight governorates in Lebanon


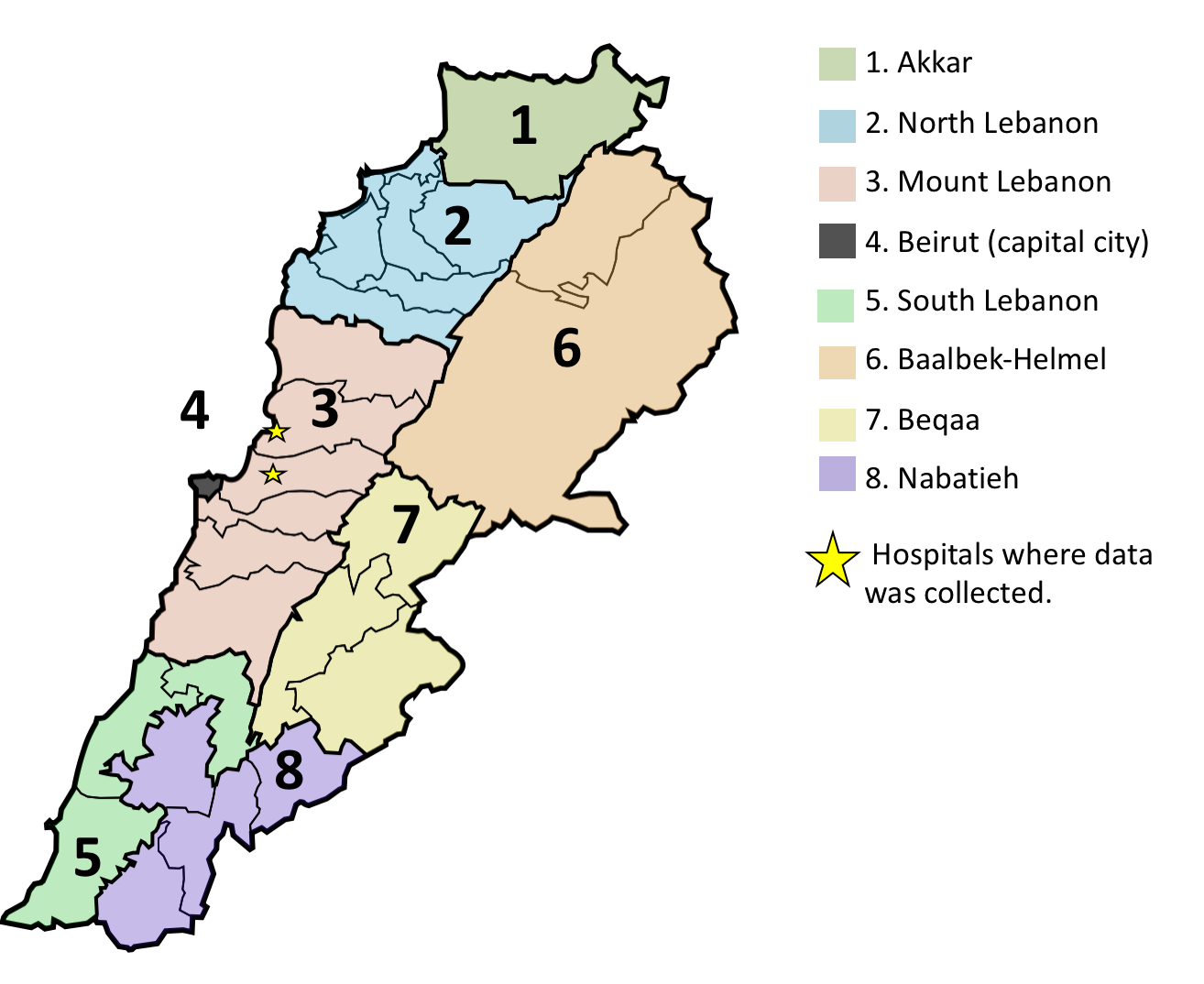


Original map was created by Crates - Own work, CC BY-SA 3.0, retrieved from <https://commons.wikimedia.org/w/index.php?curid=3560114>

**Supplementary Figure 2.** Areas of specialty of interviewed residents. Values are *n* (%). PGY, postgraduate year; OB/GYN; obstetrics and gynecology.


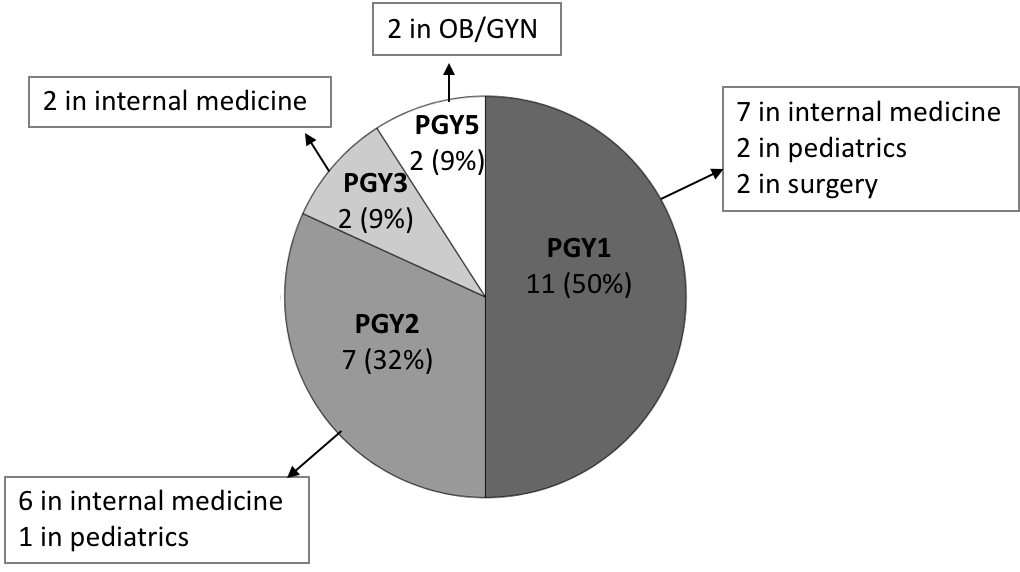


**Supplementary Table 1**. Mean scores on perceived knowledge and self-efficacy scales

|  | Interns (*n*=48) | Residents (*n*=22) | *P*-value |
| --- | --- | --- | --- |
| **Perceived knowledge**  Factor (1): Anatomy and physiology  Factor (2): Benefits of breastfeeding | 3.86 ± 0.66  4.01 ± 0.77 | 4.25 ± 0.90  4.24 ± 1.25 | 0.07  0.35 |
| **Self-efficacy**  Factor (3): Providing psycho-social support  Factor (4): Counseling about breastfeeding | 4.02 ± 0.91  3.82 ± 0.68 | 4.46 ± 0.90  4.22 ± 0.76 | 0.06  0.03 |

Values are mean ± SD. Maximum mean score per factor is 6. Differences between groups are analyzed by independent sample *t*-test.
